# Progress in Surgery

**Published:** 1898-05-21

**Authors:** 


### Progress in Surgery.

### SURGERY OF THE ŒSOPHAGUS AND STOMACH.

**"Pressure Pouches" of the Œsophagus** have been twice operated upon by Butlin.[1] The method of removal was as follows: A long incision was made in the anterior border of the left sterno-mastoid with its centre opposite the cricoid cartilage. The dissection was carried down between the trachea and œsophagus on the inner side, and the great vessels on the outer side. The superior thyroid artery was tied, the omo-hyoid muscle divided, and the pouch was discovered lying behind the œsophagus, at its junction with the pharynx, and projecting a little towards the left side. It was cut away from above downwards, while the edges were brought together with fine silk sutures. In the second case no attempt was made to keep a tube in the œsophagus for the purpose of feeding, and a drainage tube was inserted in the wound of the neck instead of strips of gauze as in the first case. Five days after operation milk came through the wound in the neck for the first time. In about eight days this had almost ceased, and three weeks after operation the wound was healed and the patient could swallow perfectly well. Before the operation is performed, while the patient is under the influence of an anæsthetic, it is desirable to pass a curved metal intrument into the pouch from the mouth, and to make its end appear in the posterior triangle of the neck, which affords the clearest evidence of the existence of a true pressure pouch.

**Œsophago Enterostomy.**—One of the most remarkable cases on record is the excision of the whole stomach by Schlatter,[2] the patient making a complete recovery. An incision was made from the ensiform cartilage to the umbilicus. The entire stomach presented itself as a hard cancerous mass extending from the cardiac to the pyloric extremity, but was freely movable. The stomach was freed from all its attachments at the greater and lesser curvature, and was forcibly dragged down so that the œsophagus might be reached. The œsophagus was clamped, and the stomach severed beneath the œsophageal extremity. Some lymph nodes were dissected out near the pyloric end, and the stomach then cut away beyond the pylorus. The duodenum was invaginated, and the opening closed by a double suture. A loop of intestine, in which a slit was cut, was then fastened to the cut end of the œsophagus.

Commenting on this case Wendt[3] forms the following conclusions: (1) The human stomach is not a vital organ. (2) The digestive capacity of the human stomach has been considerably over-rated. (3) The fluids and solids constituting an ordinary mixed diet are capable of complete digestion and assimilation without the aid of the human stomach. (4) A gain in the weight of the body may take place in spite of the total absence of gastric activity. (5) Typical vomiting may occur without a stomach. (6) The general health of a person need not immediately deteriorate on account of the removal of the stomach. (7) The most important office of the human stomach is to act as a reservoir for the reception, preliminary preparation, and propulsion of food and fluids; and it regulates the temperature of swallowed solids and liquids. (8) The chemical functions of the human stomach may be completely and satisfactorily performed by the other divisions of the alimentary canal. (9) Gastric juice is hostile to the development of many micro-organisms. (10) The free acid of normal gastric secretions has no power to arrest putrefactive changes in the intestinal tract. Hemmeter[4] refers to other cases of complete excision of the stomach—one by Bayley, for a gastric sarcoma involving all but a small portion of the stomach and the mesentery throughout the abdomen, the patient only surviving for thirty-six hours; and another by a surgeon in San Francisco, in a female patient suffering from cancer, and who was reported to be still alive, with normal pulse and temperature, thirty-six hours after operation.

Maylord[5] reports a successful case of jejunostomy by Maydl's method for carcinoma of stomach. Maydl has considerably modified the operation known as jejunostomy. The abdomen is opened in the middle line above the umbilicus, and the upper end of the jejunum is found. When this is secured, it is brought out of the abdominal wound and divided. Another opening is made in the side of the distal segment, into which the proximal is implanted. This union is effected either by stitching or by a Murphy's button. The orifice of the distal segment is lastly stitched to the parietal wound.

**Pylorectomy.**—Morison[6] suggests a new modification of connecting the remainder of the stomach to the cut duodenum. The opening into the stomach is, as would be expected, larger than the opening into the duodenum made by cutting it transversely. In order to overcome the difficulty of uniting the two without the aid of complicated sutures, an incision, over one inch in length, is made down the centre of the anterior wall of the already transversely-divided duodenum. By spreading the longitudinal cut transversely, the duodenal opening is so widened as to correspond approximately with the larger opening in the stomach. All that then remains is to connect the two by a satisfactory suture. A continuous catgut suture is employed through all the coats, and outside this interrupted silk sutures are placed through the serous and muscular coats. He supports his method by reference to the circulation of the intestine, and quotes two cases, one of which died seven weeks after operation. Jessop[7] and Hume[8] also quote successful cases of pylorectomy.

**Pyloric Stenosis** is considered by Taylor,[9] who records a case which was relieved by Loreta's operation for about a year, but was ultimately treated by posterior gastro-enterostomy. He refers to observations which have been made on the position of the stomach in the living subject, and points out that the long axis of the normal stomach is nearly vertical, and when empty the pylorus should be its most dependent part. The latter shoul lie only very slightly to the right of the middle line, and the pyloric end of the stomach should be immediately central.

**Hour-Glass Contraction of Stomach** was diagnosed in the case of a woman aged 46 by Burney Yeo and Watson Cheyne.[10] The patient had suffered for years from symptoms suggestive of ulcer of the stomach without hæmorrhage. There was more discomfort than actual pain, and vomiting occurred every second or third day, but she did not bring up nearly as much as she swallowed. The dilatation of the stomach was more marked at the cardiac than pyloric end, and gurgling could be heard as of fluid running through a narrow channel. A diagnosis of hour-glass contraction, due to an old ulcer, was made, and this was confirmed by operation. The case was successfully treated by the Heineke-Mikulicz method, being identical with that employed in cases of contracted pylorus under the name of pyloro-plasty.

**Gastric Ulcer.**—Heydenreich[11] gives the indications for surgical interference as follows: (1) In perforation it is absolutely necessary as early as possible, and to wash out the abdomen. Since 1894 the mortality has dropped to 52·94 per cent., and without operation the condition is almost necessarily fatal. (2) For stricture of the pylorus, resection is the most dangerous. Pyloro-plasty is not applicable if the ulcer extends to the pylorus, or where the pylorus is adherent, and its walls are soft and adherent. Here, perhaps, gastro-enterostomy is best, but where there is a choice pyloro-plasty is to be preferred. (3) For adhesions or abscesses in connection with the ulcer—often only diagnosed after exploratory incision. (4) For hæmatamesis. Here operation is doubtful, as sudden death is exceptional. For violent hæmorrhage laparotomy has almost always failed. For slighter hæmorrhages, which become dangerous through repetition, operation may be successful; pyloro-plasty, or more often gastro-enterostomy, have been performed with a view to procuring rest of the stomach and promoting healing of the ulcer. (5) This last consideration has led some to propose gastro-enterostomy for cases of uncomplicated gastric ulcer. The general death-rate for all cases of gastric ulcer is 25 to 30 per cent.; for gastro-enterostomy only 16·2 per cent., and therefore the operation has less danger than the disease. At any rate, cases which do not improve in a reasonable time should be treated surgically. In performing gastro-enterostomy upon a patient who apparently had pyloric stenosis from ulceration, Braun[12] found that there had been a recent rupture of the stomach. The tissues, on account of the ulcers, were so fragile that they would not retain sutures, so that the tear could not be closed by any ordinary method. He therefore brought up a fold of the great omentum and sutured it over the opening, the gastro-enterostomy was completed, the peritoneum washed out, and the abdomen closed. The patient made a good recovery. Cases of successful operation for perforating gastric ulcer are recorded by Page,[13] Toogood,[14] and Shaw.[15]

[1] Brit. Med. Journ., Jan. 1, 1898, p. 8. [2] Med. Rec., New York, Dec. 25, 1897, p. 909. [3] Ibidem, p. 913. [4] Ibidem, March 19, 1898, p. 409. [5] Lancet, Dec. 4, 1897. [6] Brit. Med. Journ., Feb. 19, 1898, p. 482. [7] Ibidem, Dec. 4, 1897, p. 1,632. [8] Lancet, Dec. 11, 1897. [9] Ibidem, Nov. 13, 1897, p. 1,235. [10] Ibidem, March 19, 1898, p. 785. [11] Epit. Brit. Med. Journ., March 26, 1898; and Sém. Med., Feb. 2, 1898. [12] Amer. Journ. of Med. Sci., Jan., 1898. [13] Lancet, April 2, 1898, p. 930. [14] Ibidem, Jan. 15, 1898, p. 158. [15] Brit. Med. Journ., March 26, 1898, p. 815.

